# Gene Therapy Tools for Brain Diseases

**DOI:** 10.3389/fphar.2019.00724

**Published:** 2019-07-01

**Authors:** Selene Ingusci, Gianluca Verlengia, Marie Soukupova, Silvia Zucchini, Michele Simonato

**Affiliations:** ^1^Department of Medical Sciences and National Institute of Neuroscience, University of Ferrara, Ferrara, Italy; ^2^Division of Neuroscience, University Vita-Salute San Raffaele, Milan, Italy; ^3^Technopole of Ferrara, LTTA Laboratory for Advanced Therapies, Ferrara, Italy

**Keywords:** gene therapy, central nervous system, viral vector, gene regulation, brain disease

## Abstract

Neurological disorders affecting the central nervous system (CNS) are still incompletely understood. Many of these disorders lack a cure and are seeking more specific and effective treatments. In fact, in spite of advancements in knowledge of the CNS function, the treatment of neurological disorders with modern medical and surgical approaches remains difficult for many reasons, such as the complexity of the CNS, the limited regenerative capacity of the tissue, and the difficulty in conveying conventional drugs to the organ due to the blood–brain barrier. Gene therapy, allowing the delivery of genetic materials that encodes potential therapeutic molecules, represents an attractive option. Gene therapy can result in a stable or inducible expression of transgene(s), and can allow a nearly specific expression in target cells. In this review, we will discuss the most commonly used tools for the delivery of genetic material in the CNS, including viral and non-viral vectors; their main applications; their advantages and disadvantages. We will discuss mechanisms of genetic regulation through cell-specific and inducible promoters, which allow to express gene products only in specific cells and to control their transcriptional activation. In addition, we will describe the applications to CNS diseases of post-transcriptional regulation systems (RNA interference); of systems allowing spatial or temporal control of expression [optogenetics and Designer Receptors Exclusively Activated by Designer Drugs (DREADDs)]; and of gene editing technologies (CRISPR/Cas9, Zinc finger proteins). Particular attention will be reserved to viral vectors derived from herpes simplex type 1, a potential tool for the delivery and expression of multiple transgene cassettes simultaneously.

## Introduction

Even if scientific research has made great progress over the last decade in identifying pathogenic mechanisms and treatment strategies, neurological disorders affecting the central nervous system (CNS) are still incompletely understood. The majority of these disorders lack a cure or, at least, reasonably effective treatments. Reasons are certainly multifold and include the complexity of the CNS, the limited regenerative capacity of the tissue, and the difficulty in conveying conventional drugs to the organ across the blood–brain barrier (BBB). Neurons, the principal cells of the nervous tissue, are not only morphologically and physiologically heterogeneous, but also strictly organized to form complex circuits. Neural stem cells ensure only a limited replacement of only specific neuronal types. The BBB expresses a selective permeability for molecules that possess a limited range of molecular weight and lipophilicity, preventing the entry of large-molecule drugs and of the majority of small-molecule drugs.

In this context, gene therapy is emerging as an attractive therapeutic option, because it can result in a stable or inducible expression of therapeutic gene(s), and can allow a nearly specific expression in target cells. Although much remains to be done before it becomes routine practice, the potential of gene therapy for the treatment of CNS diseases is amply demonstrated by numerous preclinical and clinical studies (Simonato et al., 2013).

A large part of the work needed to finally reach the stage of clinical application consists in the refinement of the tools needed for a safe, targeted, and regulated gene delivery. In this review, we will discuss the most commonly used gene therapy tools for delivery in the CNS and the strategies that can be employed for regulating therapeutic gene expression.

## Gene Therapy

The idea behind gene therapy derives from an assumption of great simplicity: by introducing into the cells the “correct” copy of a defective gene whose malfunction causes a disease, its product, a functional protein, will be able to revert the pathological phenotype. This assumption may be correct for monogenic diseases caused by alterations in the coding sequence or in regulatory regions of a single gene, and if these alterations lead to loss of function without production of a pathogenic protein. However, many diseases have a pattern of multiple altered genes. Moreover, the regulation of gene expression is often complex and difficult to reconstruct. According to a new, broader definition, all drugs that contain an active substance that includes or consists of a recombinant nucleic acid (DNA or RNA), administered to a human being for the purpose to adjust, repair, replace, add, or remove a gene sequence, can be defined gene therapy (Klug et al., 2012).

The introduction of a functional gene, called transgene, within the cell nucleus is a complex operation that starts with the choice of a delivery system (gene therapy vector). A good vector should fulfill many requirements (Kay et al., 1997; Gardlik et al., 2005; Shillitoe, 2009):

- Manipulation: the vector should be easily manipulated for recombination and propagation in suitable hosts.
- High cloning capacity: the vector should allow the introduction of one or more genes and regulatory sequences that guarantee the desired spatial and temporal restriction of transgene expression.
- Minimal invasiveness: the vector should not cause uncontrolled or undesired alterations of the host genome. The integration of a vector into the cellular genome can induce insertional mutagenesis.
- Selectivity for the cellular target: the transgene should be expressed exclusively in the target cells.
- Absence of immunogenicity: the vector should not contain genes that induce immune responses or other factors that may be harmful to the body.
- Stability over time: the vector should be transferred unaltered in the cell progeny and/or must allow a correct and prolonged expression of the transgene(s).

Available gene therapy vectors belong to two broad categories: viral and nonviral vectors.

### Nonviral Vectors

Nonviral vectors offer some advantages, like reduced pathogenicity, low cost, and simple production techniques (Zhang et al., 2004; Ramamoorth and Narvekar, 2015). However, DNA delivered by nonviral vectors must overcome a number of extracellular and intracellular barriers, limiting the efficiency of transfection (Howell et al., 2003; Matsumoto et al., 2009). In fact, the transfection efficiency is limited by the premature release of the genetic material into the bloodstream and its subsequent degradation by serum nucleases when administered intravenously, while the endocytosed DNA is in large part degraded along the endosome/lysosome pathway before it reaches the nucleus (Ogris et al., 1999; Perez-Martinez et al., 2011).

In addition to direct injection and microinjection of DNA into the nucleus, physical methods and chemical carriers have been developed to improve delivery of naked DNA to cells and tissues (Li and Huang, 2006; Ramamoorth and Narvekar, 2015). Commonly used nonviral delivery tools are cationic polymers and cationic lipids, whose efficiency is dependent on cationic charge, saturation, and linker stability (Jayant et al., 2016). Several strategies have been explored to increase the stability of DNA in the circulation. Polyethylene glycol (PEG) is one example (Dufes et al., 2005; Huang et al., 2007). Another approach is the use of bio-responsive polymers that exploit the chemical–physical properties of the biological microenvironment (pH, presence of reducing agents, etc.) to promote the release of the genetic material exclusively intracellularly. Other tested strategies are acetyl bonds, which degrade at the pH of the endosomal environment (Knorr et al., 2007; Wolff and Rozema, 2008), or disulfide bridges, which are reduced in the cytosol (Piest et al., 2008).

### Viral Vectors

Independent of their origin, order, and family, viruses have evolved very fine strategies to reach and penetrate specific cellular targets. Their use in gene therapy lies in their innate ability to deliver and express genetic information into host cells. Replication-defective viral vectors (Bouard et al., 2009) commonly derive from wild-type viruses in which the therapeutic gene(s) are inserted into the viral genome by replacing the “wild” genes essential for the lytic cycle, thereby preventing the virus to replicate and exert cytotoxic effects in target cells. These genes, however, can be replaced by trans-acting factors through the development of specific cell lines or the use of helper viruses during the manufacturing process (Zhou et al., 1996; Von Seggern et al., 1998; Morris et al., 2010). Among all viral vectors, the most characterized and used for targeting the CNS have been derived from retroviruses, adenoviruses, adeno-associated viruses, and herpes simplex viruses (Teschemacher et al., 2005; Bourdenx et al., 2014; Artusi et al., 2018). These vectors differ in payload capacity, cell tropism (**Table 1**), and ability to integrate into the host genome, which may affect the duration of transgene expression. Advantages and disadvantages, potential CNS application, and side effects for each of the above-mentioned vectors will be briefly discussed below.

**Table 1:** Main features of the most commonly employed viral vectors for CNS gene therapy.

Viral vector	Payload	Tropism
**Retrovirus vectors** Lentiviruses	up to 9 kb	Proliferating and quiescent cells
**Adenovirus vectors** 1st generation (subtype C) Helper-dependent (gutless)	7–10 kb ∼ 35 kb	Dividing and non-dividing cells Respiratory epithelium (blood cells) Highly efficient in targeting hepatocytes, less in lung, cardiac muscle, vascular neuronal tissue or dendritic cells
**Adeno-associated vectors**	∼4.8 kb	Different serotypes with different tropism; typical is tropism for hepatocytes, myocytes, and neuronal cells
**Herpes Simplex 1 vectors** Replication-competent (oncolytic) Replication-defective Amplicon vectors	∼40 kb 30–50 kb up to 150 kb	Actively dividing tumor cells like glioblastoma, hepatocellular carcinoma or melanoma cells Nondividing neuronal cells Neurons, glial cells, epithelial cells

#### Retroviral/Lentiviral Vectors

The *Retroviridae* family consists in a broad range of small RNA viruses whose common feature is to replicate through a DNA intermediate. γ-Retrovirus and lentivirus belong to this family (Cooray et al., 2012). The former is more suitable for *ex vivo* gene therapy applications, because it does not efficiently infect nondividing cells and because it is difficult to reach high viral titers (Gardlik et al., 2005). The latter infects both proliferating and quiescent cells, ensuring long-term gene expression in the absence of inflammatory responses (Case et al., 1999; Sutton et al., 1999; Sakuma et al., 2012).

While the lentiviral integrative nature ensures stable and persistent expression of the transgene, it also entails the risk of insertional mutagenesis. However, gene editing allowed to develop safe lentiviral vectors with specific integration sites (Lombardo et al., 2007). To increase the safety of lentiviral vectors, the viral genome can be split in multiple plasmids, thereby making recombinant virus generation very unlikely (Milone and O’Doherty, 2018). In addition, envelope glycoproteins (gp) can be pseudotyped to redirect viral particles to specific targets (Yee et al., 1994; Aiken, 1997; Sinclair et al., 1997; Maurice et al., 2002; Verhoeyen et al., 2003).

Since lentiviral vectors transduce neurons effectively, they have been tested for the treatment of Alzheimer’s disease (AD) and Parkinson’s disease (PD) (Azzouz et al., 2002; Jarraya et al., 2009; Palfi et al., 2014; Katsouri et al., 2016; Palfi et al., 2018).

#### Adenoviral Vectors

Adenoviruses are linear double-stranded DNA viruses with a genome size of 35–40 kb encoding approximately 30–40 genes. There are 100 serotypes of adenovirus, 57 of which have the potential to infect humans. These are divided into seven subgroups, A to G, that differ in cellular tropism (Khare et al., 2011; Lee et al., 2017). The most frequently utilized for gene therapy are types 2 and 5 (Campos and Barry, 2007), both belonging to subtype C (Chang et al., 2008).

Adenoviral vectors transduce efficiently dividing and nondividing cells, with no risk of integration in the host cell genome (Lee et al., 2017). Their main limitations are high immunogenicity; transient transgene expression (from 2 weeks to a few months); and high risk of cytopathic effects (Christ et al., 1997; Ritter et al., 2002; Lowenstein and Castro, 2003; Lowenstein and Castro, 2004; Cucchiarini, 2016). New generations of adenoviral vectors partially overcome these limitations (Lowenstein and Castro, 2002; Gardlik et al., 2005; Campos and Barry, 2007).

Adenoviral vectors have been widely studied for the treatment of tumors (Dobbelstein, 2004; Rein et al., 2006; Sharma et al., 2009; Lenman et al., 2018). In addition, preclinical studies have been conducted in rodent models of PD and Huntington disease (HD) (Choi-Lundberg et al., 1998; Bemelmans et al., 1999).

#### Adeno-Associated Vectors

Adeno-associated viruses (AAVs) are small, non-enveloped, single-stranded DNA viruses belonging to the *Parvoviridae* family. Despite the size limitation, they are considered the most promising vehicle for gene therapy targeting the CNS because they are clinically safe and effective in transducing dividing and quiescent cells, while capable of establishing a long-term transgene expression. More than 150 clinical trials with a good safety profile and significant clinical benefit in many genetic diseases used AAV vectors (Penaud-Budloo et al., 2018). The AAV genome contains only three genes, replication (*rep*), assembly (*aap*), and capsid (*cap*) (Naso et al., 2017), necessary for viral replication, integration, and packaging (Berns and Giraud, 1996; Nakai et al., 1999; Musatov et al., 2002). AAVs can persist in the host cell in an episomal state, only a small fraction integrating into the host cell genome (Bouard et al., 2009).

More than 12 different AAV serotypes have been isolated (Duan, 2016). Each of these has specific features, including: differences in cellular tropisms (Nakai et al., 2005; Mandel et al., 2006), depending on different capsid surface proteins (Kaludov et al., 2001; Wu et al., 2006; Bell et al., 2011; Shen et al., 2011); differences in transduction efficiency; and differences in ability to evade the host immune response and to cross the BBB (Zhang et al., 2011; Yang et al., 2014). Many serotypes transduce efficiently neurons and glial cells (Davidson et al., 2000; Wang et al., 2003a; Burger et al., 2004; Aschauer et al., 2013). Refinement of AAV vector features and increased brain uptake have been obtained by pseudotyping approaches, like mixing of capsids and genome from different viral serotypes (Grimm et al., 2008; Mao et al., 2016; Hammond et al., 2017); insertion of peptide motifs from phage libraries (Chen et al., 2009); or enrichment of the capsid through random incorporation of peptide motifs (Choudhury et al., 2016; Deverman et al., 2016; Korbelin et al., 2016; Chan et al., 2017).

Owing to the small sized genome, AAV vectors are capable of accommodating less than 5 kb of exogenous DNA (Wang et al., 2014; Chira et al., 2015). However, strategies are under development for delivering larger genes. One approach could be using truncated forms of genes and/or promoters that maintain the properties of their full-length counterpart (Wang et al., 2000; Kodippili et al., 2018; Zhang et al., 2018). An alternative strategy has been developed by taking advantage of the AAV innate ability to undergo a genomic intermolecular recombination that can give rise to head-to-tail DNA concatamerization (Yan et al., 2005). In this context, the DNA sequence of an oversized expression cassette can be split and packaged in two (Duan et al., 1998; Yan et al., 2000; Duan et al., 2001; Ghosh et al., 2008; Trapani et al., 2014) or even three (Maddalena et al., 2018) independent AAV vectors. Upon concomitant infection of the host, these multiple vectors may give rise to DNA concatamers, which can reconstitute the whole cassette (Maddalena et al., 2018). The efficiency of this multiple-vector strategy is obviously significantly lower than the single-vector strategy (Duan et al., 2001), and attempts are ongoing to improve the situation (Ghosh et al., 2008).

The therapeutic potential of AAV-based gene therapy has been tested in many different neurological disorders (Kaplitt et al., 2007; Eberling et al., 2008; Worgall et al., 2008; Christine et al., 2009; Souweidane et al., 2010; Mittermeyer et al., 2012; Rafii et al., 2014; Tardieu et al., 2014; Mendell et al., 2017).

#### Herpes Simplex Vectors

The first Herpesvirus vector has been derived from Herpes Simplex Virus type 1 (HSV-1) (Palella et al., 1988), an enveloped ubiquitous virus with a double-stranded linear DNA genome. Hallmarks of HSV-1 are the short replication cycle and the ability to travel *via* retrograde axonal transport from the primary infection site to the sensory neurons of the CNS, where it establishes a life-long latency in an episomal form (Studahl et al., 2017). Since then, three types of HSV-1 based vectors have been developed: replication-competent, replication-defective, and amplicon vectors (Artusi et al., 2018). Replication-competent vectors are used in oncology for their ability to complete a lytic cycle in the presence of permissive cellular environments (Ikeda et al., 2000; Todo, 2008). Replication-defective and amplicon vectors are tested as gene transfer tools for the CNS (Goverdhana et al., 2005; Berges et al., 2007).

The large genome (152 kb) of HSV-1 encodes about 80 genes, half of which can be removed to make room for up to 50 kb of foreign DNA in the case of replication-defective vectors and up to 150 kb for amplicon vectors (Simonato et al., 2000). HSV-1 vectors maintain high infectivity, ability of both retrograde and anterograde transport, and potential to establish a latent infection in the episomal form (Lachmann, 2004). Traditional limitations in their application are residual toxicity towards the infected cells (Goverdhana et al., 2005) and the short-term expression of the transgene due to rapid silencing mechanisms (Lachmann et al., 1996).

### New HSV-Based Vectors for Delivering Multiple Expression Cassettes

Disorders of the CNS are often not a result of single gene mutation or of a single molecular mechanism but have instead a multifactorial origin. As a result, the therapeutic gene(s) and/or the regulation sequence to be delivered very often exceed the payload capacity of viral vectors (Thomas et al., 2003). HSV-1 vectors represent an attractive solution for this issue, because of their large genomic size and capacity to host large amounts of foreign DNA. As noted above, however, the first generations of replication-defective HSV-1 vectors were hindered by highly significant limitations, including toxicity and short-term expression of the transgenes (Marconi et al., 1999). Many recent studies have been aimed to solve these problems.

**Figure 1:**
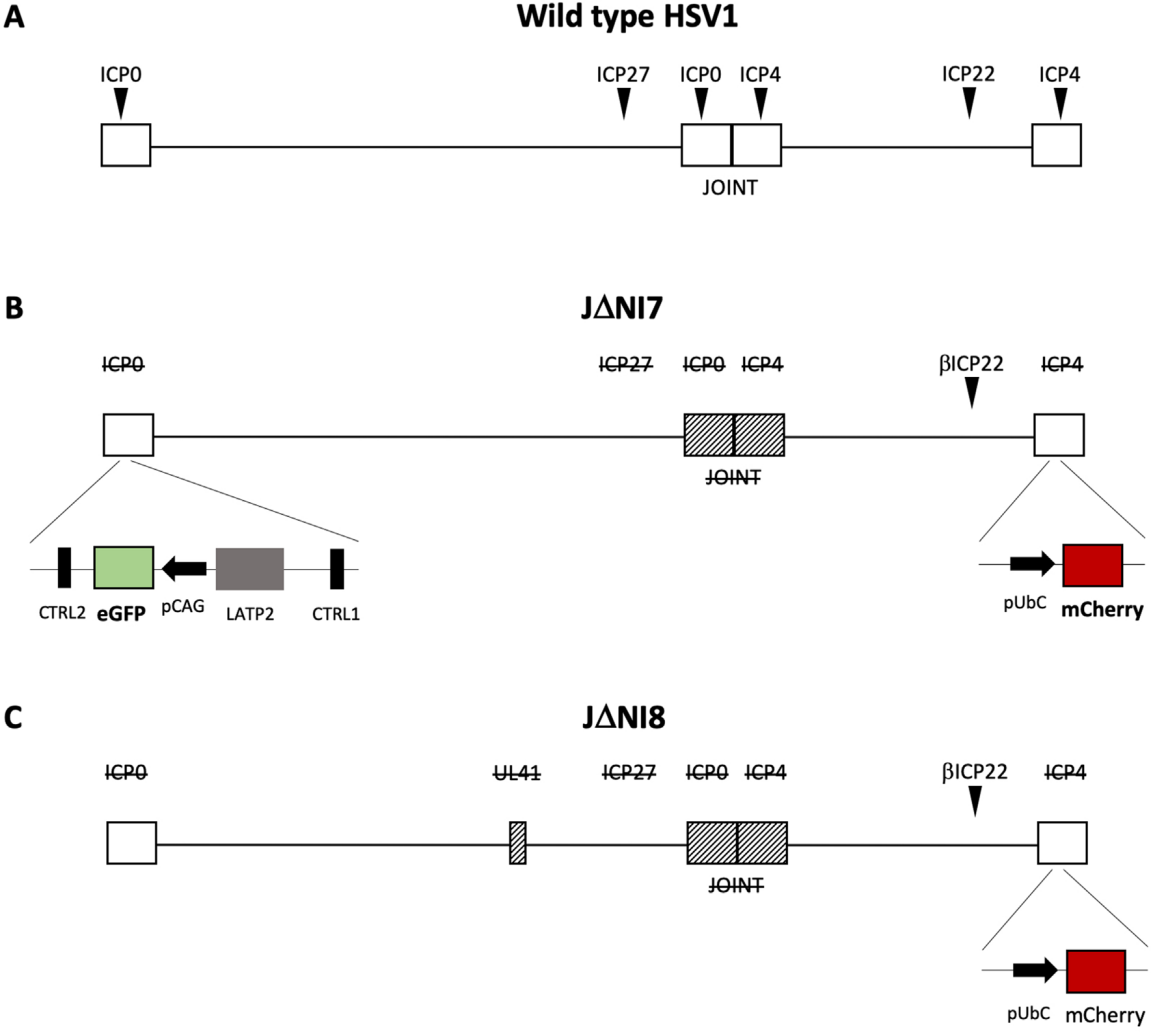
Schematic drawing of the genomes of new HSV-1 vectors. Compared to the wild-type genome **(A)**, the derivative vectors JDNI7 **(B)** and JDNI8 **(C)** are deleted for the joint region and the ICP0, ICP4, and ICP27 immediate-early genes, while the ICP22 immediate-early gene is converted to early-expression kinetics by promoter modification. Moreover, both viral backbones are engineered for the insertion of a ubiquitin C promoter (UbCp)-mCherry cassette within the ICP4 locus. The JDNI7 **(B)** backbone is further modified by the insertion of a CAG promoter-eGFP expression cassette into the LAT (Latency Associated Transcript) intron region, flanked by the LATP2 enhancer region, and CTCF-binding motifs (CTRL2). The last generation of HSV-based vectors, JDNI8 **(C)**, is also deleted for UL41, the gene encoding the virion host shutoff (vhs) protein.

The HSV productive cycle is characterized by a temporally regulated cascade of gene expression, during which three distinct classes of transcripts are expressed in a sequential manner. The immediate early genes (IE or α) are first expressed, followed by the early (E or β) and late (L or γ) genes. IE genes are required not only for establishment of a lytic reproductive cycle, but also to overcome innate immune responses, to block cell division, and to prevent host cell apoptosis and epigenetic repression of viral genes. The engineering of mutant HSV-1 vectors devoid of α genes shuts off viral replication and remarkably reduce cytotoxicity (Krisky et al., 1998). Deletion of two IE genes, ICP4 and ICP27, gave rise to the first (ΔICP4) and second (ΔICP4/27) generation of HSV-1 vectors (Shepard and DeLuca, 1991; Wu et al., 1996). These early generations were able to establish a long-term expression without the ability to reactivate, but the residual presence of ICP0 was responsible of cytotoxic effects in transduced cells. The consequent deletion of ICP0 gave rise to a third generation of vectors that were devoid of toxicity but displayed short-term expression of the transgene (Samaniego et al., 1997). To overcome this hurdle, a novel generation of HSV-1 vectors (**Figure 1**) was engineered by inserting the transgene expression cassette into the viral latency-associated transcript locus, a genome region that is protected from silencing during latency by the presence of insulator sequences (CTRL) that act as boundary elements to shield the locus against epigenetic modifications (Bloom et al., 2010). This new generation of highly defective vectors offers a large payload capacity while displaying no sign of toxicity upon *in vitro* infection of diverse cell types (Miyagawa et al., 2015). The *in vivo* injection of this new class of HSV-based vectors into different brain regions of naive rats yielded a robust and persistent (up to 6 months) neuron-specific expression of transgenes inserted in the ICP4 locus, without evidence of toxicity or inflammatory cell infiltration, suggesting that the ICP4 locus may be an option to achieve a sustained and prolonged transgene expression in neurons (Verlengia et al., 2017). A further modification of the viral backbone (the deletion of the UL41 gene that may contribute residual toxicity) further improved the levels of transgene expression. UL41-deleted vectors displayed an improved, long-lasting and neuron-specific transgene expression without any evidence of toxicity (Miyagawa et al., 2017). Recently, in order to overcome the rapid transcriptional repression of transgenes cloned outside the ICP4 locus, the backbone was further engineered with specific cellular anti-silencing elements, inserted to prevent the formation of heterochromatin at the transgene promoter level. Different types of anti-silencing elements were evaluated, but the most effective in increasing neuronal transgene expression in both *in vitro* and *in vivo* assays was A2UCOE (Han et al., 2018). In conclusion, these new replication-defective backbones seem to hold the features and the flexibility of options needed for gene therapy applications in the CNS.

**Figure 2:**
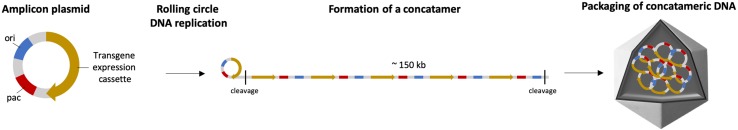
Schematic representation of an amplicon vector. The amplicon plasmid contains the sequences for viral DNA replication (ori, blue) and the packaging signal (pac, red), flanking the transgene expression cassette. In the presence of an HSV-1 helper virus in permissive cells, the amplicon DNA plasmid is replicated as head-to-tail concatemers, cleaved into 150 kb linear DNA and packaged in HSV capsids.

Amplicon vectors (**Figure 2**) are the other promising class of HSV-1-based vectors. Amplicons maintain all structural, immunological, and host range properties of the wild-type virus, but only two elements, the viral origin of replication (*ori*) and the capsid packaging sequence (*pac*), are retained from their original genome, the other part consisting of a concatemer repetition of the foreign DNA (Epstein, 2009b). The major advantage of this gene transfer tool is that the total deletion of viral genes allows the insertion of up to 150 kb of exogenous DNA. The number of concatameric repeats depends on the size of the expression cassette, i.e., short sequences allow the insertion of more copies of the gene of interest. Furthermore, the complete absence of all viral genes strongly reduces the risk of reactivation, complementation, or recombination with latent HSV-1 genomes (Epstein, 2009a). The downside is that amplicon propagation is rather difficult, because cell lines able to complement all viral proteins in trans are not available. Therefore, first generation of amplicon vectors was propagated by transfection of amplicon plasmids into cells, which were then superinfected with an HSV-1 helper virus. Unfortunately, this approach leads to a significant (about 1%) contamination with helper virus (Pechan et al., 1996). A promising alternative is the use of the LaL helper virus, in which the packaging sequence can be deleted by Cre-lox specific-site recombination, reducing the helper contamination to 0.05–0.5% (Zaupa et al., 2003). Despite the fact that amplicon vectors have been widely used for exploring the mechanisms of CNS function, only few studies have focused on their use for gene therapy (Cuchet et al., 2007). Recently, ultrapure amplicons produced using a highly defective helper virus have been employed to silence brain-derived neurotrophic factor (BDNF) expression in an animal model of epilepsy (Falcicchia et al., 2016). Apart from the production difficulties, amplicon vectors suffer from a relatively short-time expression of the transgenes in the living organism. Most likely, the presence of bacterial sequences in the amplicon genome causes transgene silencing by forming inactive chromatin (Suzuki et al., 2006). Therefore, the current research in this field focuses on the development of new, helper virus independent production techniques and on means to obtain long-term expression of the transgene.

## Cargo

### Genes Encoding Therapeutic Proteins

The question that comes together with the development of suitable vectors for gene transfer in the CNS is which genes to transfer. The first, most obvious option is genes encoding a therapeutic protein. This may be the correct copy of a defective gene whose malfunction causes the disease, but also a gene encoding a therapeutic protein that could not be peripherally administered, not being able to cross the BBB. In this last instance, the protein could be diffusible (i.e., produced and secreted by the infected cell) to produce a by-stander effect in adjacent cells (Simonato, 2014).

Apart from genes encoding therapeutic proteins, however, there are other cargo options for CNS gene therapy vectors, like gene editing, chemogenetic, and optogenetic tools.

### Gene Editing Tools

A step forward was made in gene therapy with the development of gene editing tools that can correct genetic defects directly in the host DNA. These tools generate a double strand break (DSB) at a precisely desired location, and the break allows to take advantage of the fine strategies that cells have evolved to detect and repair DNA damage. The DSB can lead to gene disruption by non-homologous end joint (Lieber, 2010), or gene addition or repair by homologous recombination using an exogenously supplied repair template (Li and Heyer, 2008). The most promising gene editing system is the Clustered Regularly Interspaced Short Palindromic Repeats/Cas (CRISPR/Cas) system (Gaj et al., 2016; Ahmad et al., 2018). The delivery platforms described in this review are obviously essential for this new and powerful therapeutic tool.

### Chemogenetic and Optogenetic Tools

The complexity of the mammalian brain has no comparison: dozens of billions of interconnected neurons, with complex morphology and circuit interaction, capable of exchanging electrical signals with a precise temporal scan in the order of milliseconds. A great challenge is to develop the ability to control only one type of cell in the brain without affecting others. Electrical, physical, pharmacological, and genetic methods are traditionally used to manipulate cells and synapses (Carter and Shieh, 2015). However, all these methods lack temporal and spatial resolution and can cause stimulation, inhibition, or inactivation of off-target cells and processes. To overcome these limitations, new genetic tools referred to as “chemogenetic” and “optogenetics” have been developed.

#### Designer Receptors Exclusively Activated by Designer Drug

Chemogenetics is the processes in which proteins are engineered to interact specifically with a small molecule (Sternson and Roth, 2014). Different proteins involved in CNS disorders have been engineered to this aim, including enzymes (Bishop et al., 1998; Cohen et al., 2005; Dar et al., 2012), and G protein-coupled receptors (GPCRs; Zemelman et al., 2003; Magnus et al., 2011). The latter include allele-specific GPCRs (Strader et al., 1991), Receptors Activated Solely by a Synthetic Ligand (RASSLs; Coward et al., 1998), and Designer Receptors Exclusively Activated by Designer Drugs (DREADDs; Armbruster et al., 2007). The first chemogenetically engineered GPCR was the β-adrenergic receptor (Strader et al., 1991). Following mutations at the β-adrenergic receptor binding site, the responsiveness to the natural ligand disappeared and a new specificity was obtained to ketonic and catechol esterase agonists. Several years later, RASSLs receptors were developed, based on human κ-opioids, that lost their affinity for the endogenous peptide ligand (dynorphin) and gained specificity for small and safe drugs such as spiradoline (Coward et al., 1998).

Together with optogenetics, the DREADDs technology is currently the most used tool for *in vivo* manipulation of the activity of genetically defined neuronal populations. An example of DREADDs technology is the use of modified muscarinic receptors, hM3Dq for stimulation and hM4Di for inhibition, which have lost their affinity for endogenous acetylcholine, but can be activated by a synthetic ligand (clozapine-N-oxide, CNO) that crosses the BBB (Armbruster et al., 2007; Alexander et al., 2009). Compared to previous techniques, chemogenetics based on DREADDs confers the ability to regulate and manipulate in a non-invasive manner the activity of specific neuronal circuits. Combined with the set of gene therapy tools described above, DREADDs can be delivered and almost selectively expressed in the neuronal subpopulation of interest, for example serotoninergic (Teissier et al., 2015; Urban et al., 2016) or glutamatergic neurons (Krashes et al., 2014; Zhu et al., 2014; Zhu et al., 2016).

DREADDs are useful tools for basic scientific research but may also refine gene therapy approaches for neurodegenerative disorders in which changes in neuronal activity play an important role. Neuronal hyperactivity and hyperexcitability of the cerebral cortex and hippocampus are common features of epilepsy and AD (Badawy et al., 2009; de Haan et al., 2017). On demand attenuation of seizures was achieved after delivery of AAV vectors carrying the hM4Di receptor under the control of the *CamkIIα* promoter (Katzel et al., 2014). Moreover, transient cholinergic-specific stimulation led to a striking improvement in motor scores in a rodent model of PD including gait and postural abnormalities (Pienaar et al., 2015).

#### Optogenetic Approaches

The term optogenetics indicates a methodology that allows to control the activity of specific neurons within intact neuronal circuits (Deisseroth, 2011). The idea of using the light as a tool to control neuronal function was originally put forward by Francis Crick (Crick, 1979). In the 1970s, biologists discovered that some microorganisms generate proteins that, in response to visible light, regulate the flow of charges across the membranes (Oesterhelt and Stoeckenius, 1971). These proteins, termed opsins, are photosensitive trans-membrane proteins that, when illuminated at defined frequencies, induce transmembrane ion fluxes and, thereby, changes in the electrical activity of the cell. There are two major classes of opsins, which differ in sensitivity to light and absorption properties, and cause activation or inhibition of neurons (Deisseroth, 2015):

- Channelrhodopsin (ChR): a blue light activated cation-channel from *Chlamydomonas reinhardtii*, used to excite neurons (Nagel et al., 2003);
- Halorhodopsin (NpHR): a yellow light activated chloride-pump from *Natronomonas pharaonic*, used to inhibit neurons (Chow et al., 2010).

Through viral vectors, the gene coding for an opsin can be integrated into target neurons, leading to expression of the opsin protein on the membrane. A nearby source of light, set on the right wavelength and frequency, can then interact with it, activating or inhibiting neuronal activity. The introduction of mutations to existing opsin variants allowed to overcome certain problems associated with light delivery. For example, the ChR chETA mutant displays faster channel closing and increased temporal control (Gunaydin et al., 2010). The C1V1 mutant can be excited by longer wavelengths, allowing deeper penetration of the tissue (Yizhar et al., 2011). At the same time, light delivery tools have been improved and optimized for *in vivo* studies (Liu et al., 2015).

The use of optogenetics as therapeutic tools for neurological disorders has been investigated in PD, AD, and epilepsy (Kravitz et al., 2010; Zahedi et al., 2013; Seeger-Armbruster et al., 2015). Enhanced excitation of pyramidal neurons is a common feature of many forms of epilepsy, and its control may lead to therapeutic effects. NpHR delivery into these neurons promptly and dramatically reduced seizures upon light stimulation. Enhancing inhibitory activity of interneurons *via* transfection of the excitatory opsin ChR also resulted in reduced seizure frequency and severity upon light stimulation (Krook-Magnuson et al., 2013). Optical inhibition of the subthalamic nucleus in PD models significantly improved akinesia and ameliorated levodopa-induced dyskinesia (Yoon et al., 2014; Yoon et al., 2016).

## Regulation of Gene Expression

As previously mentioned, precise regulation of gene expression is essential for any gene therapy approach. A good gene regulation system should be adjustable over a broad dose range; should exert no off-target effect; does not influence endogenous gene expression; should be region or cell speciﬁc; and should allow to quickly turn off and on transgene expression (Naidoo and Young, 2012). Regulation of gene expression, in terms of increasing or decreasing levels of a specific gene product or in terms of directing the information to a desired target tissue, can occur at different levels. In this section, we will discuss the gene regulation strategies currently used in gene therapy, e.g., at the transcriptional level, by means of tissue specific and inducible promoters; and at post-transcriptional level, by means of RNA interference techniques.

### Transcriptional Level: Tissue Specific and Inducible Promoters

Regulation or RNA transcription depends on the euchromatin and heterochromatin state and on the interaction of transcription factors with regulatory DNA elements, including promoters (**Table 2**), insulators, enhancers, and silencers.

**Table 2:** Regulation of gene expression at transcriptional level.

Promoter	Specificity	Size (bp)	Details	References
CAG	Ubiquitous	1,718	Hybrid construct consisting of the cytomegalovirus (CMV) enhancer fused with the chicken beta-actin promoter	(Qin et al., 2010)
EF1α	Ubiquitous	1,179	Human elongation factor 1 alpha promoter	(Qin et al., 2010)
UBC	Ubiquitous	1,177	Human ubiquitin C promoter	(Qin et al., 2010)
SV40	Ubiquitous	627	Simian virus 40 promoter	(Qin et al., 2010)
CMV	Ubiquitous	589	Human cytomegalovirus immediate early enhancer and promoter	(Qin et al., 2010)
PGK	Ubiquitous	511	Mouse phosphoglycerate kinase 1 promoter	(Qin et al., 2010)
Syn1	Neuron	495	Human synapsin 1 promoter	(Kugler et al., 2001; Kugler et al., 2003; McLean et al., 2014)
NSE	Neuron	1,800	Neuron-specific enolase promoter	(Peel et al., 1997)
GFAP	Astrocytes	681–2,200		(Smith-Arica et al., 2000; Lee et al., 2008)
MAG	Oligodendrocytes	1,500–2,200	Human myelin associated glycoprotein	(von Jonquieres et al., 2013)
MBP	Oligodendrocytes	1,900	Myelin basic promoter	(von Jonquieres et al., 2016)
F4/80	Microglia	667		(Rosario et al., 2016)
CD68	Microglia	460		(Rosario et al., 2016)
PAG	Glutamatergic neurons	2,400	Phosphate-activated glutaminase promoter	(Rasmussen et al., 2007)
vGLUT	Glutamatergic neurons	7,000	Vesicular glutamate transporter promoter	(Rasmussen et al., 2007)
GAD	GABAergic neurons	10,000	Glutamic acid decarboxylase promoter	(Rasmussen et al., 2007)
Tetracycline ON/OFF system	Inducible promoter		Advantages: rapid *in vivo* induction or inhibition kinetics, low toxicity. Limitations: high basal transgene expression.	(Gossen et al., 1995; Xiong et al., 2006)
Rapamycin regulation system	Inducible promoter		Advantages: low basal expression, trigger by low doses, crosses BBB. Limitations: immunosuppressive properties of rapamycin.	(Rivera et al., 1996)

#### Ubiquitous and Tissue-Specific Promoters

Promoters are the main elements that determine the strength and cellular specificity of gene expression. Ubiquitous and constitutive promoters are strongly active in a wide range of cells and tissues. Therefore, ubiquitous expression promoters are used in gene therapy when targeting a specific cell type is not required, i.e., transgene expression is sought in the broadest possible spectrum of cells. Promoters frequently employed to drive exogenous DNA expression in a non-cell specific manner include cytomegalovirus (CMV) immediate-early; enhancer/chicken-β actin (CAG); human ubiquitin C (UBC); simian virus 40 early (SV40); human elongation factor 1α (EF1α); and mouse phosphoglycerate kinase 1 (PGK). Previous works have described the relative strengths of commonly used transcriptional regulatory elements both *in vitro* and *in vivo* (Pasleau et al., 1985; Martin-Gallardo et al., 1988; Oellig and Seliger, 1990; Yew et al., 1997; Manthorpe et al., 1993; Hartikka et al., 1996). CAG, EF1α, and CMV are the strongest among those analyzed. However, the CMV promoter exerts variable results, being very strong in some cell types and rather weak in others (Qin et al., 2010).

The use of cell-type specific promoters may be useful for confining the transgene expression to a specific cell type. Limitations for their use in gene therapy include their low level of expression and their large genomic size. In principle, however, specifically labeling a population of neurons or glial cells might allow to achieve the therapeutic goal without incurring in off-target effects. The synapsin-1 (Syn1) and the neuron-specific enolase (NSE) promoter are used for their ability to selectively drive transgene expression in neurons (Peel et al., 1997; Kugler et al., 2001; Kugler et al., 2003; McLean et al., 2014), while the glial fibrillary acidic protein (GFAP) promoter results in astrocyte-specific expression (Smith-Arica et al., 2000; Lee et al., 2008). Transgene expression can be specifically targeted to oligodendrocytes by the myelin basic protein (MBP) (von Jonquieres et al., 2013) or the human myelin associated glycoprotein (MAG) promoter, the latter in both a full-length and a truncated version (von Jonquieres et al., 2016). High level of microglial cells specificity can be obtained with the F4/80 promoter (Rosario et al., 2016).

The balance between excitatory and inhibitory signals, basically the equilibrium between glutamatergic and GABAergic neurotransmission, can be often disrupted in diseases like epilepsy. Therefore, targeting specifically GABAergic or glutamatergic neurons may be needed for many applications. The phosphate-activated glutaminase (PAG) or the vesicular glutamate transporter (vGLUT) promoter ensures ∼90% glutamatergic neuron-specific expression, whereas the glutamic acid decarboxylase (GAD) promoter ensures ∼90% GABAergic neuron-specific expression (Rasmussen et al., 2007).

Promoters are not the only elements necessary for transcriptional regulation. Combining regulation elements of different kinds such as promoters, enhancers, introns, and polyadenylation signals by creating hybrid sequences allows modulation of the expression levels. The levels of transgene expression may be strongly influenced by a rapid epigenetic silencing of the exogenous promoters. To protect the promoter and the whole expression cassette from heterochromatization, insulator elements have been tested for their ability to maintain transcriptionally competent whole portions of DNA, regardless of the tissue type and the integration site (Chung et al., 1993; Bell et al., 1999). As reviewed extensively elsewhere, these protective elements are divided into enhancer-blocking insulators, whose function is mediated by the CTCF-binding factor (Gaszner and Felsenfeld, 2006; Phillips and Corces, 2009) and barrier insulators, whose mechanism of action is less known (West et al., 2002; Gaszner and Felsenfeld, 2006; Raab and Kamakaka, 2010). The first well-characterized vertebrate insulator derived from the chicken β-globin locus associated with a constitutive DNAse I, the hypersensitive site-4 called cHS4 (Chung et al., 1993). cHS4 exhibits both enhancer-blocking activity and barrier activity (Yao et al., 2003). Many studies concerning the delivery of retroviral vectors showed that the inclusion of the cHS4 element allows to increase transgene expression (Rivella et al., 2000; Emery, 2011). Others, such as the ubiquitous chromatin opening element (UCOE) derived from the human HNRPA2B1-CBX3 locus (A2UCOE), show increased stability of transgene expression due mainly to resistance to DNA methylation-mediated gene silencing (Zhang et al., 2010). Other elements to consider include enhancer elements, whose insertion significantly affects the expression levels of the transgene (Hartikka et al., 1996). Often used in genetic engineering is the CMV enhancer (eCMV), whose presence in cultured cells strongly increases the expression of the transgene when under the control of PDGF-β (platelet-derived growth factor-β) promoter, conferring efficient neurons specific gene expression (Yew et al., 1997; Gruh et al., 2008).

#### Inducible Promoters

For many applications, it is desirable to modulate the expression of the transgene by switching it on or off. Unregulated long-term overexpression of certain transgenes can cause side effects in the CNS, such as aberrant reorganization of the tissue and activation of compensative pathways and/or inactivation/saturation of activated pathways. A finer regulation can be achieved using inducible promoters. These systems are obtained by incorporating in the vector (or in a separate vector) a cassette driving the constitutive expression of a transcription factor (transactivator) able to activate or block the expression of the transgene depending on the availability of a soluble molecule that can be administered systemically.

##### The Tet On/Tet Off Regulation System

**Figure 3:**
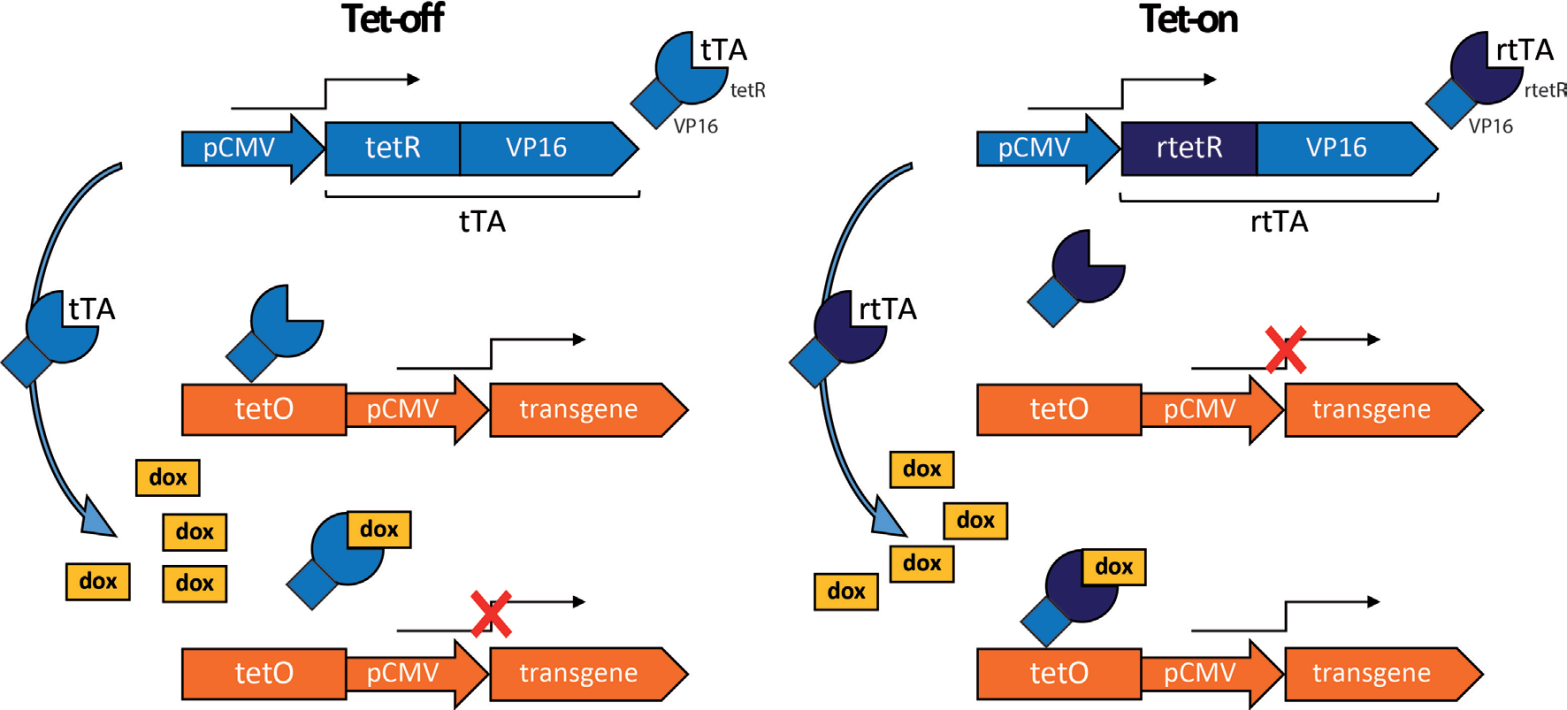
Tetracycline regulation system. In this example, the constitutively active human cytomegalovirus promoter (pCMV) drives the expression of the tetracycline transactivator (tTA) or of the tetracycline reverse transactivator (rtTA), consisting respectively of the tet-repressor (tetR) or reverse tet-repressor (rtetR) fused to the VP16 transactivation domain. Tet-off: tTA binds the tet operator (tetO) to drive transgene expression in the absence, but not in the presence of doxycycline (dox). Tet-on: rtTA binds to tetO and drives transgene expression in the presence, but not in the absence of dox.

A commonly used regulation system is based on the mechanism of tetracycline resistance in prokaryotes. Two variants are available (**Figure 3**), both relying on tetracycline to deactivate (Tet-off system) or activate gene expression (Tet-on). In the Tet-off system, the transgene is under the transcriptional control of the tet operator (tetO), a fragment of DNA responsive to the transactivator tTA, composed of the tet-repressor (tetR) fused to the VP16 viral protein transactivation domain. The transgene is expressed only when tTA binds tetO in the absence of doxycycline, an analogue of tetracycline. Otherwise stated, the presence of tetracycline or its analog in the culture medium or in the organism reversibly induces the transactivator to detach from the operator, causing the transgene to “switch off.” The Tet-on system was developed by inducing random mutations in the tetR. One mutation resulted in a protein with opposite function, which was named reverse tet-repressor (rtetR). This mutant protein, when fused to the VP16 transactivation domain (reverse transactivator, rtTA), drives transgene expression only in the presence of doxycycline (Gossen et al., 1995).

Tetracycline-based regulatory systems hold a great potential for gene therapy applications. They ensure rapid *in vivo* induction or inhibition kinetics (Xiong et al., 2006) and low toxicity—tetracycline and its derivatives have been used for decades for their antimicrobial activity (Pasquale and Tan, 2005). Limitations are mainly due to high basal transgene expression, in particular when the expression is driven by a constitutive promoter. Recently, a second generation of tetracycline-regulated system containing a shortened CMV minimal promoter was found to increase regulation efficiency and display low basal expression (Agha-Mohammadi et al., 2004). Modifications in the tetO sequence (TRE-tight1) driven by an NSE were found to improve gene expression efficiency and to reduce the leaky basal transcription (Tian et al., 2009).

For CNS applications, it is necessary to obtain adequate concentrations of doxycycline in the brain, which is difficult due to the limited ability of this drug to cross the BBB (Nau et al., 2010). However, new rtTA variants containing mutations in the doxycycline contact domain enhance sensitivity to doxycycline, resulting in a reduction of the concentrations required for transgene regulation (Das et al., 2004; Zhou et al., 2006). The majority of CNS applications involving the tetracycline-regulated system used viral vectors for the construct delivery, e.g., lentiviruses (Georgievska et al., 2004; Pluta et al., 2005), adenoviruses or AAVs (Ebert et al., 2005; Lee et al., 2005; Le Guiner et al., 2014), and resulted in a tightly regulated gene expression. For example, the lentiviral vector-mediated delivery of GDNF regulated by a Tet-on system in a PD rat model resulted in a precise regulation of transgene expression and in neuroprotection of nigral DA neurons (Chen et al., 2014).

##### The Rapamycin Regulation System

**Figure 4:**
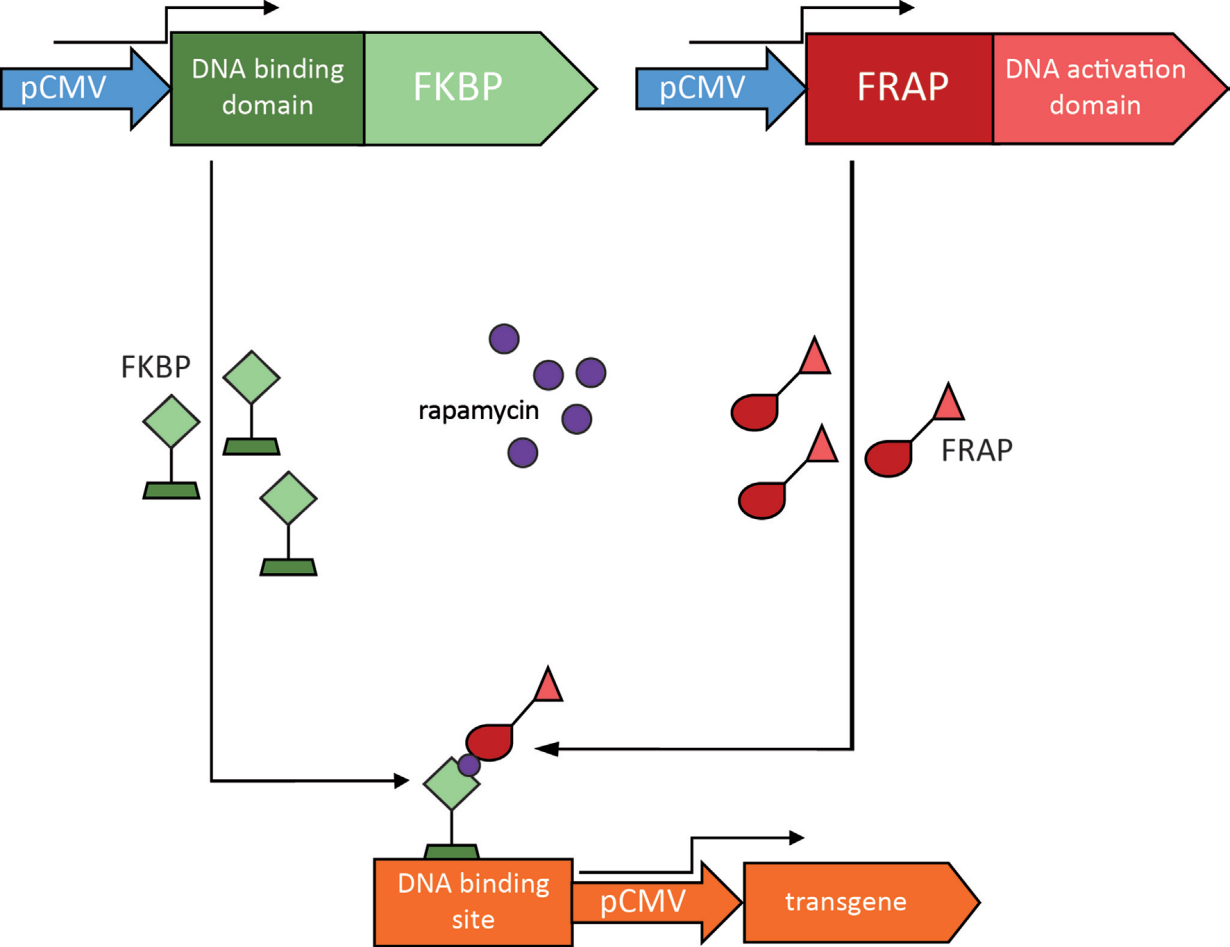
Rapamycin regulation system. In this example, the constitutively active human cytomegalovirus promoter (pCMV) drives the expression of two transcription factors, one consisting of the FK-binding protein (FKBP) fused to a DNA binding domain (in green), the other consisting of an FKBP12 rapamycin-associated protein (FRAP) fused to a DNA activation domain (in red). Rapamycin enables dimerization of the transcription factors and the resulting heterodimer is able to drive expression of the transgene of interest.

The rapamycin regulation system is based on the interaction between two inactive transcription factors, a DNA binding domain and a DNA transcriptional activation domain. Each transcription factor is fused to heterologous binding domains for rapamycin. The DNA binding domain is fused to three copies of the FK-binding protein (FKBP), while the DNA activation domain is fused to a lipid kinase, FKBP12 rapamycin-associated protein (FRAP) (Rivera et al., 1996; Kang et al., 2008). Rapamycin allows their interaction, thus forming an active, heterodimeric transcription factor that drives the expression of the transgene (**Figure 4**).

This system holds many of the features required for clinical use. First, rapamycin is a clinically approved drug, used as an antifungal and antitumor molecule that can be administered orally and can cross the BBB (Dutta et al., 2014). Second, the system ensures low basal expression of the transgene in the absence of rapamycin, but can be triggering by low doses of the drug (Naidoo and Young, 2012). The limitations are mainly related to the immunosuppressive properties of rapamycin, dependent on blockade of the mTOR signaling pathways. The rapamycin analogues “rapalog” (e.g., AP21967) were engineered by adding substituents that prevent the binding to mTOR (Bayle et al., 2006).

A limited number of studies tested this system in the CNS by delivering it through lentiviral (Vogel et al., 2008), AAV (Sanftner et al., 2006), and HSV-1-based vectors (Wang et al., 2003b). A dose-dependent response to rapamycin was observed after delivering into the rat striatum a rapamycin regulated AAV2-GDNF vector, by evaluating GDNF biogenesis and accumulation under various rapamycin dosing regimens. In addition, chronic administration with clinically compatible regimens of rapamycin provided GDNF protein levels similar to those reported to be neuroprotective in PD animal models (Hadaczek et al., 2011).

### Post-Transcriptional Gene Regulation

**Figure 5:**
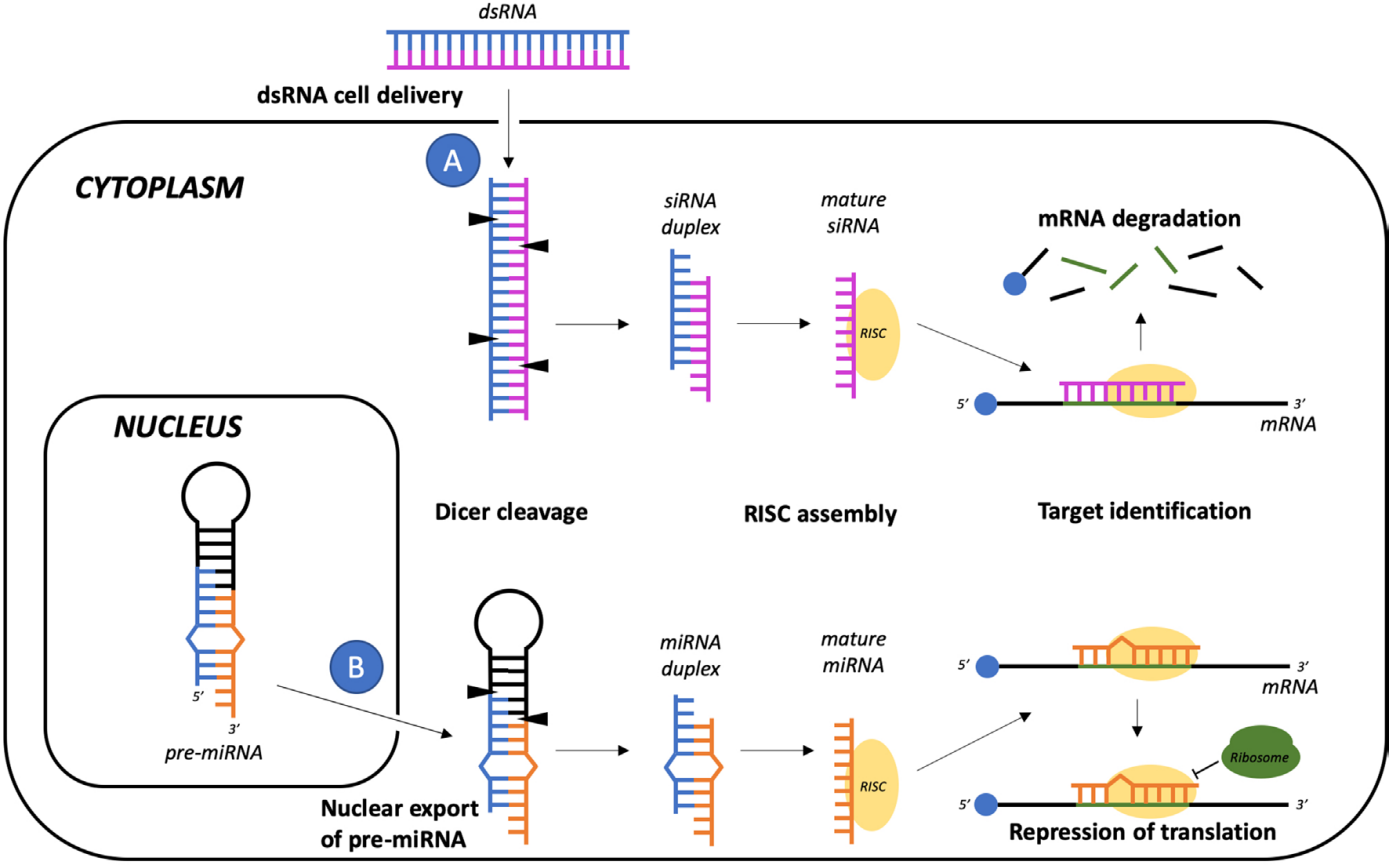
RNA interference-gene silencing pathways mediated by siRNA or miRNA. Even though siRNAs precursors are commonly delivered into the target cells as exogenous double-stranded RNAs **(A)** while the pre-miRNA hairpin structure is exported from the nucleus after nuclear transcription and processing **(B)**, in the cytoplasm these small RNA sequences both share common pathways of maturation and target mRNA recognition, which respectively involve DICER cleavage and association of one strand of the duplex with the RNA-induced silencing complex (RISC). Both the siRNA-RISC and the miRNA-RISC complexes may then sequence-specifically hybridize the target mRNA, leading to mRNA degradation or to translation repression.

The term “post-transcriptional gene regulation” refers to approaches designed to enhance degradation or block translation of a target mRNA. The prototypical example is RNA interference (RNAi, **Figure 5**), a physiological and evolutionarily conserved gene silencing mechanism normally present in eukaryotic cells, which is mediated by a group of noncoding small RNAs (Mattick and Makunin, 2006). Although many types of small RNAs have been identified, the main classes seem to be short interfering RNAs (siRNAs), microRNAs (miRNAs), and piwi-interacting RNAs (piRNAs) (Gomes et al., 2013). Guided by the interaction with Argonaute proteins, these small RNAs can mediate the degradation or the inhibition of translation of a specific mRNA, making it possible to fine-tune gene expression (Hock and Meister, 2008). In 2018, the US Food and Drug Administration approved Patisiran, the first ever RNAi therapy licensed for clinical use. Patisiran is a formulation of lipid nanoparticles that can be intravenously administered to deliver siRNAs designed to suppress the production of transthyretin, which aggregates into amyloid fibrils that cause nerve damage in patients with hereditary transthyretin amyloidosis (Coelho et al., 2013; Adams et al., 2018).

Neurodegenerative diseases resulting from a single gene mutation that causes gain-of-function or accumulation of a mutant protein are potential candidates for RNAi. Unfortunately, RNA molecules do not cross the BBB, thus requiring the use of viral or nonviral vectors for CNS delivery. This may result in widespread changes in expression levels in unrelated genes due to nonspecific degradation of nontarget mRNAs (Jackson et al., 2003; Persengiev et al., 2004; Scacheri et al., 2004) and saturation of endogenous silencing pathways (Grimm et al., 2006).

For example, RNAi has been investigated in HD, an autosomal-dominant neurodegenerative disorder caused by a CAG trinucleotide repeat expansion in the huntingtin (HTT) gene (Kremer et al., 1994). A potential therapeutic strategy for HD is to reduce mutant HTT expression by using shRNA or miRNA molecules against HTT mRNA. Using RNAi against HTT mRNA does not imply the down-regulation of only the mutated protein, but also of the wild-type protein that plays an important role in neuronal survival (Reiner et al., 2003). To reduce off-target effects, a series of bioinformatic programs have been developed to predict the hypothetical target mRNAs for each specific miRNA. These programs take into account target complementarity, evolutionary conservation among species, and the thermodynamic stability of the heteroduplex formed by the interaction between miRNAs and mRNAs (Reynolds et al., 2004). Several studies have shown a significant reduction in HTT mRNA together with improved motor function in mouse models of HD upon brain delivery of AAV vectors encoding for shRNA designed to target HTT mRNA (Harper et al., 2005; Rodriguez-Lebron et al., 2005). However, evidence of side effects prompted new studies using artificial miRNAs for gene silencing. This approach improved the therapeutic effect in the absence of any detectable detrimental effect up to 9 months after treatment (Boudreau et al., 2009; Drouet et al., 2009), and has been employed in many subsequent preclinical studies not only on HD (Miniarikova et al., 2016; Pfister et al., 2017; Pfister et al., 2018) but also on superoxide dismutase 1-linked amyotrophic lateral sclerosis (Foust et al., 2013; Borel et al., 2018).

**Figure 6:**
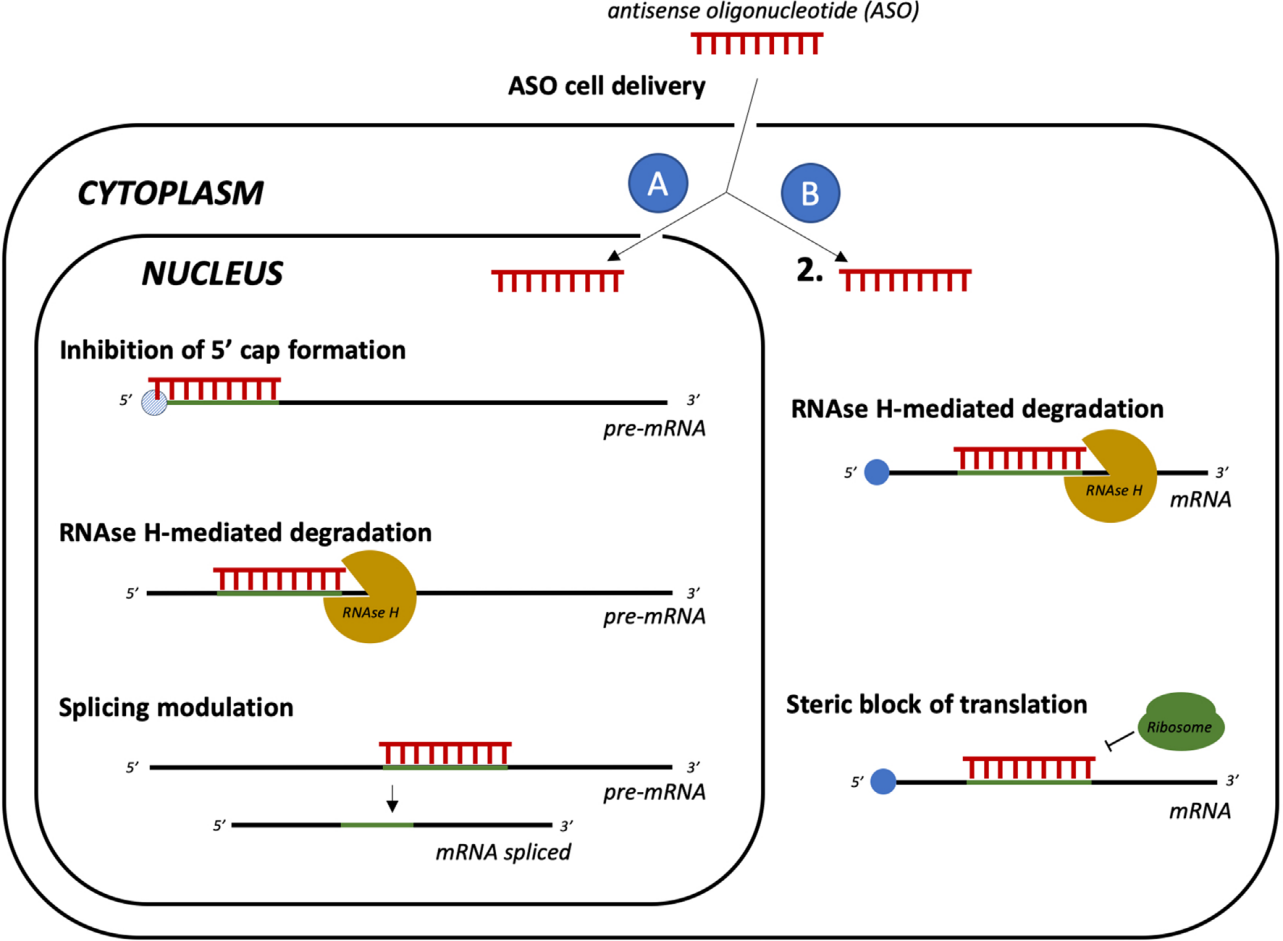
Gene silencing by delivery of antisense oligonucleotides (ASO). Cell-delivered ASOs can act as translation repressors with different mechanisms. If ASOs reach the nucleus **(A)**, they can form heteroduplexes with the pre-mRNA, which can result in inhibition of 5’ cap formation, recruitment of RNAse H, and mRNA degradation. They may also even act as splicing modulators. In the cytoplasm **(B)**, ASOs can hybridize with the target mRNA, leading to RNAse H-mediated degradation or to steric block of ribosome binging, resulting in impaired translation.

New promising tools acting at a post-transcriptional level are short antisense oligonucleotides (ASO, **Figure 6**) with RNA/DNA-based structures that can sequence-specifically hybridize RNA, turning it into a target for RNase H-mediated degradation (Wu et al., 2004). The main advantages of ASOs are a higher affinity to target compared with small RNAs, which results in decreased or null off-target toxicity, and the ability to cross the cell membrane to bind RNAs in the cytoplasm or even in the nucleus (Geary et al., 2015). ASOs have been intensively tested in human neurodegenerative disorders. For example, ASOs that selectively decrease human tau mRNA have been shown to reduce tau protein deposition and neuronal loss in a mouse model of AD (DeVos et al., 2017). Moreover, ASOs developed for targeting the mutated form of HTT (mHTT) are currently in clinical development for HD. Initial results with a non-allele specific ASO indicate absence of toxicity and reduced levels of mHTT (Tabrizi et al., 2019). Clinical trials with allele-specific ASOs for mHTT are also ongoing (Potkin and Potkin, 2018).

## Conclusions

The rapid progress of viral and nonviral vector systems has increased the probability of success of CNS gene therapy as an alternative to existing pharmacological treatments. However, all the delivery systems developed thus far have advantages and disadvantages and, therefore, the search for an ideal one continues. A lesson learned from the research performed to date is that delivery tools do not necessarily adapt to all applications, but should be chosen according to the specific situation and need. Understanding the rules of transcriptional and post-transcriptional gene regulation will allow to improve our techniques. In addition, optogenetic and chemogenetic approaches can provide a precise temporal and spatial regulation of gene expression, and the recent introduction of genome-editing technologies allows the direct manipulation of the genome. Therefore, even if more work will be needed to overcome the remaining hurdles, gene therapy now holds a strong promise to become a safe and effective option for CNS diseases in the not too distant future.

## Author Contributions

SI, GV, and MSi wrote the largest part of the article. MSo wrote the chapter on amplicons, and SZ wrote the chapter on viral vectors.

## Funding

This work was supported by grants from the European Community (FP7-PEOPLE-2011-IAPP project 285827 [EPIXCHANGE] and FP7-HEALTH project 602102 [EPITARGET]).

## Conflict of Interest Statement

The authors declare that the research was conducted in the absence of any commercial or financial relationships that could be construed as a potential conflict of interest.
